# Primary Tumor Characteristics as Biomarkers of Immunotherapy Response in Advanced Melanoma: A Retrospective Cohort Study

**DOI:** 10.3390/cancers16132355

**Published:** 2024-06-27

**Authors:** Rachel S. Goodman, Seungyeon Jung, Kylie Fletcher, Hannah Burnette, Ismail Mohyuddin, Rebecca Irlmeier, Fei Ye, Douglas B. Johnson

**Affiliations:** 1 Vanderbilt University School of Medicine, Nashville, TN 37240, USAkylie.a.fletcher@vanderbilt.edu (K.F.); rebecca.irlmeier@vumc.org (R.I.); fei.ye@vumc.org (F.Y.); 2 Department of Hematology/Oncology, Vanderbilt University Medical Center, Nashville, TN 37240, USA; hannah.r.burnette@vumc.org; 3 Vanderbilt University, Nashville, TN 37240, USA; ismail.a.mohyuddin@vanderbilt.edu; 4 Department of Biostatistics, Vanderbilt University Medical Center, Nashville, TN 37240, USA

**Keywords:** melanoma, immunotherapy, biomarkers

## Abstract

**Simple Summary:**

Melanoma survival rates have been vastly improved by the use of immune checkpoint inhibitors. However, some patients do not respond to the treatment or experience progression of disease. Because of this variability of response, markers predicting efficacy of these treatments is of interest. This study aimed to clarify the role of primary tumor characteristics in melanoma treatment and survival. The authors demonstrated that cutaneous melanoma and its subtypes were significantly associated with response, progression-free survival, and overall survival compared with acral or unknown primary melanoma. Other primary features did not demonstrate an association on multivariable analyses. Thus, primary features, other than cutaneous primary, should likely not influence metastatic treatment selection.

**Abstract:**

Identifying patients likely to benefit from immune checkpoint inhibitor (ICI) treatment remains a crucial goal for melanoma. The objective of this study is to assess the association between primary tumor features and immunotherapy response and survival in advanced melanoma patients. In this single-center retrospective cohort study, disease characteristics, response to immunotherapy, PFS, and OS were assessed among melanoma patients (excluding mucosal and uveal primaries) treated with ICI. Among 447 patients, 300 (67.1%) received anti-PD-1 monotherapy and 147 (32.9%) received ipilimumab/nivolumab. A total of 338 (75.6%) had cutaneous melanoma, 29 (6.5%) had acral melanoma, and 80 (17.9%) had melanoma of unknown primary. Ulceration and stage at initial presentation were associated with inferior outcomes on univariate analysis. However, on multivariate analysis, this result was not observed, but cutaneous melanoma and each of its subtypes (superficial spreading, nodular, other, unknown) were positively associated with response, longer PFS, and longer OS. Metastatic stage (M1c, M1d) at presentation (OR = 1.8, *p* < 0.05) and *BRAF*^V600E^ mutation status (OR = 1.6, *p* < 0.001) were associated with shorter PFS. This study is limited by its retrospective and single-center design. Cutaneous melanoma and its subtypes were significantly associated with response, PFS, and OS compared with acral or unknown primary melanoma.

## 1. Introduction

The advent of immune checkpoint inhibitors (ICI) targeting cytotoxic T-lymphocyte-associated antigen 4 (CTLA-4) and programmed death 1 (PD-1) has transformed the management of advanced melanoma. Five-year survival has increased from 5% historically to >50% with combination immunotherapy [1,2]. While many patients exhibit durable responses, approximately half experience progression of the disease [3,4,5]. The variability in response remains unclear.

Despite the growing use of ICI across tumor types, there is a lack of well-defined prognostic or predictive markers of ICI efficacy and survival. While efforts have been made to identify factors associated with response (and lack thereof), such as tumor factors, immune microenvironment, and host factors [6,7,8,9,10,11,12], the impact of primary tumor characteristics on therapy response is unclear. One study found that primary melanoma thickness, ulceration, and stage were associated with brain metastases [13]. Thus, the primary tumor characteristics could reflect the underlying disease biology (e.g., hypoxia, immune response, aggressiveness) that impacts other aspects of metastatic behavior including the immunotherapy response. Another study demonstrated that primary melanomas located on sun-exposed skin treated with first-line anti-PD1 therapy had improved progression-free survival (PFS) compared to those on intermittently sun-exposed areas or sun-protected areas [14].

In this single-center retrospective cohort study, we assessed clinical features, primary tumor characteristics, response, and survival outcomes in 447 patients with unresectable or metastatic melanoma treated with ICI.

## 2. Methods

### 2.1. Overview

We conducted a single-center retrospective cohort study to assess the characteristics of primary melanomas and ICI outcomes. The study was approved by the Vanderbilt University Medical Center institutional review board. Patients receiving one or more doses of anti-PD-1 monotherapy or combination of ipilimumab and nivolumab were included. Patients with melanoma of mucosal or uveal origin were excluded due to their distinct tumor biology and published lower responses to anti-PD-1 [15,16,17].

### 2.2. Data Collection

Retrospective clinical data were collected, including date of initial diagnosis, anatomic site of primary, subtype (cutaneous, acral, or unknown primary), histologic subtype (superficial spreading [SSM], nodular, other, unknown), Breslow thickness, ulceration status (0/1), number of mitoses, presence of lymphovascular invasion (LVI), and tumor-infiltrating lymphocytes (TILs). The “other” cutaneous histologic subtype category aggregated multiple subtypes including desmoplastic, spindle cell, lentigo maligna, amelanotic, spitzoid, and balloon cell. Pre-immunotherapy treatment details, *BRAF* and other mutation status, number of positive sentinel nodes (0, 1–2, 3+), M status, and stage (AJCC 8th edition [18]) after the staging workup were collected. LDH level and stage (AJCC) at the time of immunotherapy initiation were collected. Immunotherapy outcomes were evaluated according to the therapeutic response (RECIST criteria), progression details, and date of last follow-up, which was defined as the date of last survival follow-up or death, whichever occurred later.

### 2.3. Statistical Analyses

Descriptive statistics were used to summarize patient and treatment characteristics. Univariate analyses assessed the association between each primary tumor characteristic and the response to ICI, progression-free survival (PFS), and overall survival (OS). Categorical variables and continuous variables were analyzed using a chi-squared test and a Wilcoxon rank-sum test, respectively. PFS and OS were tested using log-rank tests. Continuous variables were summarized by mean, median, and interquartile range (IQR). To estimate the independent effect of each clinical variable on the outcome, we conducted multivariable analyses using logistic regression for the response and Cox regression for the PFS and OS. In the Cox models, variables that did not meet the proportional hazard assumption were treated as time-dependent covariates, allowing their effect on the outcome to change over time. The multivariate analyses results were reported as odds ratios (ORs) or hazard ratios (HRs) with 95% confidence intervals (CIs). A backwards selection identified the independent variables that were most strongly associated with the outcome to avoid overfitting and improve model interpretation. To assess the impact of treatment, a reduced model without treatment status was compared to the full model using a likelihood ratio test. All of the statistical analyses were conducted using R v4.2.2.

## 3. Results

### 3.1. Patient and Primary Tumor Characteristics

A total of 447 patients (137 female [30.6%]; mean [SD] age, 60.9 [14.6] years) were included. Of these, 300 (67.1%) patients received anti-PD-1 monotherapy, and 147 (32.9%) patients received ipilimumab and nivolumab. *BRAF*^V600E^ mutations were present in 140 (31.3%) of patients, and 177 (39.6%) patients had elevated LDH at the start of treatment.

In total, 338 (75.6%) patients had cutaneous melanoma, 29 (6.5%) had acral melanoma, and 80 (17.9%) had melanoma of unknown primary (Table 1). Among the patients with cutaneous melanoma, 107 (23.9%) had SSM, 76 (17.0%) had nodular, 29 (6.5%) had another subtype, and 126 (28.2%) were unknown (subtype not specified, but cutaneous primary confirmed). “Other” cutaneous subtypes included lentigo maligna (N = 11, 37.9%), desmoplastic (N = 8, 27.6%) and spindle cell (N = 8, 27.6%). At initial presentation, the mean [SD] Breslow thickness was 4.2 mm [5.3], ulceration was present in 137 (30.6%) patients, the mean [SD] mitotic count was 6.5/mm^2^ [6.7], and LVI and TILs were present in 169 (37.8%) patients and 128 (28.6%) patients, respectively. One-hundred ninety (42.5%) patients had localized disease, 125 (27.9%) had regional disease, and 110 (24.5%) had metastatic disease at presentation.

An objective response rate (ORR; complete or partial response) occurred in 128 (42.7%) patients treated with anti-PD-1 and 69 (46.9%) for those who received ipilimumab and nivolumab (Table 2). The mean PFS [SD] was 19.3 months [26.5] for anti-PD-1, and 20.2 months [25] for the combination therapy. The mean OS [SD] was similarly comparable—31.1 months [30.9] for anti-PD-1 versus 28.7 months [25.1] for the combination therapy.

### 3.2. Univariate Analysis

#### 3.2.1. Response to ICI

The melanoma site of origin, ulceration, number of positive sentinel nodes, and the stage at presentation showed significant associations with the ORR (Table 3). By site of origin, the ORR was 44.7% (151/338) for cutaneous, 10.3% (3/29) for acral, and 36.7% (29/79) for unknown primary (*p* < 0.01). The ORRs by subtype were as follows: 39.4% for SSM (43/109), 53% for nodular (41/78), and 55% for other types (16/29). The ORR in patients with ulceration was 35.8% (49/137) and 51.0% (79/155) for those without ulceration. The ORR was 53.2% (82/154) for those with 0 positive sentinel nodes, 28% (27/95) for 1–2 positive sentinel nodes, and 39% (12/31) for 3+ positive sentinel nodes (*p* < 0.01). For the stage, the ORR was 49.1% (86/175) for patients who initially presented with stage I/II melanoma and 35.3% (96/272) for patients presenting with stage III/IV melanoma (*p* < 0.01). The Breslow thickness (*p* = 0.3), mitoses (*p* = 0.5), LVI (*p* = 0.5), TILs (*p* = 1), and M status at presentation (*p* = 0.56) were not associated with the response.

#### 3.2.2. Progression-Free Survival

**Figure 1:**
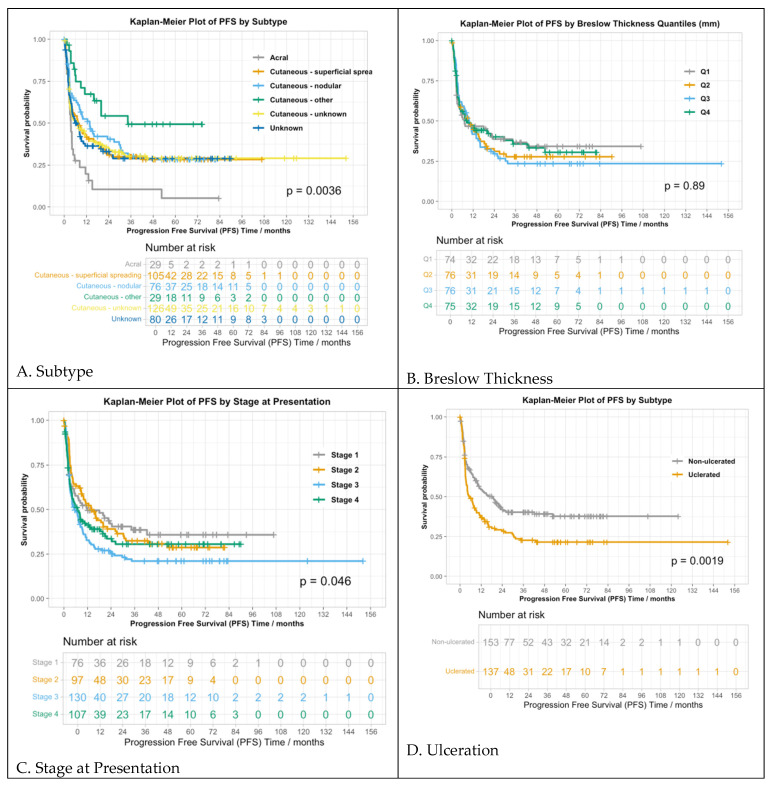
Primary tumor characteristics and progression-free survival. Progression-free survival by (**A**) subtype of melanoma; (**B**) Breslow thickness; (**C**) stage at presentation; (**D**) ulcerated vs. non-ulcerated.

Among all patients, the median PFS was 8.2 months (95% CI: 5.8–10.4, N = 445), 3.6 months for patients with acral melanoma (95% CI: 2.7–5.6), 7.6 months for SSM (95% CI: 4.3–16.2), 13.8 months for nodular (95% CI: 8.1–30.3), 34.3 months for other cutaneous subtype (95% CI: 16.02–NA), 7.2 months for unknown cutaneous subtype (95% CI: 3.6–13.1), and 7 months for unknown primary (95% CI: 4.1–11.3) (*p* < 0.01) (Figure 1A). The patients were categorized into four quartiles based on their Breslow thickness: Q1 (<1.4 mm), Q2 (1.4–2.7 mm), Q3 (2.7–5 mm), and Q4 (>5 mm) (Figure 1B). There was no significant difference in the PFS among the quartiles, with PFSs of 7.2, 9.6, 9.7, and 8.9 months for Q1–4, respectively (*p* = 0.9). The stage at initial disease presentation was associated with the PFS, with median PFSs of 11.8, 14.0, 5.6, and 7 months for stages I, II, III, and IV, respectively (*p* = 0.05) (Figure 1C). For the M status, the overall difference in the PFS was not statistically significant (*p* = 0.3), with median PFSs of 8.6, 18.4, 24.4, 5.3, and 5.7 months for M0, M1a, M1b, M1c, and M1d, respectively. The median PFSs were 5.2 months (95% CI: 3.7–9.4) and 18.4 months (95% CI: 10.0–26.2) in those with and without ulceration, respectively (*p* < 0.01) (Figure 1D). Other factors associated with PFS (or with nearly significant trends) included the number of positive sentinel nodes (*p* < 0.01), mitoses (*p* = 0.07), primary presentation (*p* = 0.06), and BRAF mutation (*p* < 0.01), whereas LVI (*p* = 0.1) was not associated with PFS.

#### 3.2.3. Overall Survival

**Figure 2:**
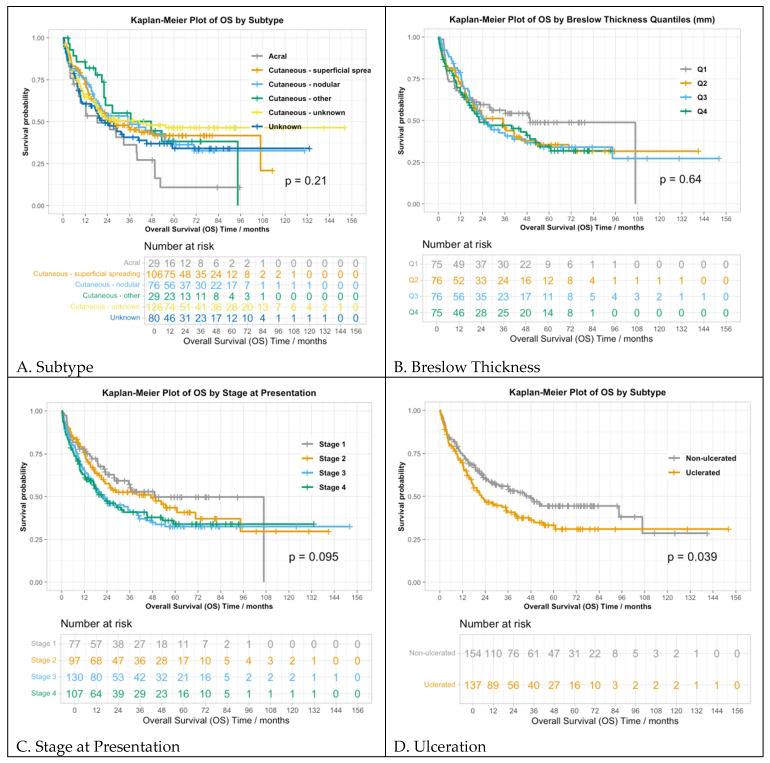
Primary tumor characteristics and overall survival. Overall survival by (**A**) subtype of melanoma; (**B**) Breslow thickness; (**C**) stage at presentation; (**D**) ulcerated vs. non-ulcerated.

The median OS for all patients was 28 months (95% CI: 21.9–41.1), with 247 out of 446 patients (55%) having died before last follow-up. As with PFS, the OS was different between those with ulceration (21.9 months, 95% CI: 17.0–39.7) and without ulceration (47.7 months, 95% CI: 29.2–NA) (*p* = 0.02). Similarly, OS varied by M stage, with median OS values of 35.1, 45.3, not reached, 16.9, and 9.6 months for those with M0, M1a, M1b, M1c, and M1d, respectively (*p* = 0.04) (Figure 2D). The number of positive sentinel nodes was also associated with OS (*p* < 0.01).

OS analysis by subtype did not reveal statistically significant differences, with median OS values of 18.6, 27.4, 36.1, 47.7, and 41.1 months for acral, superficial spreading, nodular, other cutaneous, unknown cutaneous subtypes, and unknown subtype, respectively (*p* = 0.21) (Figure 2A). Moreover, the association between the stage at presentation and OS was not statistically significant (*p* = 0.10): the median OSs were 49.1, 47.2, 21.0, and 21.9 months for stages I-IV (Figure 2C). Other factors not associated with OS included LVI (*p* = 0.6), TILs (*p* = 0.1), Breslow thickness (*p* = 0.64), and BRAF mutation (*p* = 0.19) (Figure 2B).

### 3.3. Multivariable Analysis

#### 3.3.1. Response to ICI

The multivariable analyses with backward selection revealed that gender, TILs, treatment, Breslow thickness, stage at presentation, M status, LVI, age, prior therapy, ulceration, mitoses, and elevated LDH were not significantly associated with the response to ICI (*p* > 0.05). In the reduced logistical regression model, the superficial spreading subtype (OR = 6.5, *p* < 0.01), nodular subtype (OR = 10.5, *p* < 0.01), other cutaneous subtype (OR = 9.0, *p* < 0.01), unknown cutaneous subtype (OR = 7.4, *p* < 0.01), and unknown subtype (OR = 7.1, *p* < 0.01) were associated with the response (reference group: acral). The *BRAF*^V600E^ mutation (OR = 0.4, *p* < 0.01) and 1–2 positive sentinel nodes (reference group: 0 positive sentinel nodes; OR = 0.50, *p* < 0.01) were negatively associated with the response. When treatment was added to the model, it was not a significant predictor of the therapeutic response (OR = 1.1, *p* = 0.7) and did not significantly affect the model likelihood ratio test or discrimination indexes. We also wondered whether the loss of association of ulceration with response was due to a more aggressive metastatic presentation in patients with ulceration. Ulceration was associated with higher metastatic stage at presentation with a marginally significant *p*-value (*p* = 0.06) but not with higher LDH (*p* = 0.45) (Table 4).

#### 3.3.2. Progression-Free Survival

The backwards selection removed the number of positive sentinel nodes, TILs, Breslow thickness, age, LVI, and treatment from the model (*p* > 0.05). The reduced cox regression model showed that SSM (OR = 0.6, *p* < 0.05), nodular subtype (OR = 0.5, *p* < 0.05), other cutaneous subtype (OR = 0.3, *p* < 0.001), unknown cutaneous subtype (OR = 0.5, *p* < 0.05), unknown primary (OR = 0.5, *p* < 0.05), and male gender (OR = 0.7, *p* < 0.05) were associated with longer PFS. Metastatic stage (M1c, M1d) at presentation (OR = 1.8, *p* < 0.05) and *BRAF*^V600E^ mutation status (OR = 1.6, *p* < 0.001) were associated with shorter PFS. The presence of ulceration, mitoses, initial presentation stage (II, III, M1a, M1b), elevated LDH, and prior therapy were not associated with PFS.

#### 3.3.3. Overall Survival

The number of positive sentinel lymph nodes, TILs, gender, LVI, Breslow thickness, treatment status, ulceration, mitoses, and M status were removed in the model selection for OS (*p* > 0.05). SSM (OR = 0.6, *p* = 0.03), other cutaneous subtype (OR = 0.5, *p* < 0.05), unknown cutaneous subtype (OR = 0.5, *p* < 0.01) were associated with longer OS while age (OR = 1, *p* < 0.001), prior therapy (OR = 1.1, *p* < 0.05), and *BRAF*^V600E^ mutation status (OR = 1.4, *p* < 0.05) were associated with shorter OS. The treatment type did not have an independent effect (*p* = 0.5).

**Table 4:** Multivariable analysis: response to anti-PD-1, PFS, and OS.

Response to Anti-PD-1			
Variable	Odds ratio	95% CI	*p*-value
SSM subtype	6.50	1.8–23.45	0.004
Nodular type	10.48	2.850–38.50	<0.001
Other cutaneous type	8.97	2.13–37.71	0.003
Unknown subtype	7.36	2.06–26.32	0.002
Unknown primary	7.07	1.90–26.33	0.004
1–2 positive sentinel nodes	0.50	0.30–0.83	0.008
3+ positive sentinel nodes	0.68	0.32–1.41	0.300
BRAF mutation	0.38	0.24–0.61	0.001
PFS			
Variable	Odds ratio	95% CI	*p*-value
SSM subtype	0.58	0.36–0.94	0.026
Nodular type	0.53	0.33–0.86	0.010
Other cutaneous type	0.31	0.15–0.61	<0.001
Unknown subtype	0.55	0.34–0.88	0.013
Unknown primary	0.50	0.28–0.88	0.016
Ulceration	1.24	0.92–1.66	0.162
Mitoses	0.99	0.97–1.01	0.202
Stage II at presentation	1.14	0.73–1.78	0.561
Stage III at presentation	1.42	0.93–2.16	0.103
Stage IV M1a/b at presentation	0.94	0.53–1.68	0.842
Stage IV M1c/d at presentation	1.79	1.07–3.00	0.027
Male gender	0.75	0.58–0.96	0.022
Prior treatment	1.04	0.91–1.20	0.542
LDH > ULN	0.92	0.79–1.06	0.236
BRAF mutation	1.64	1.27–2.11	<0.001
OS			
Variable	Odds ratio	95% CI	*p*-value
SSM subtype	0.58	0.35–0.95	0.029
Nodular type	0.65	0.39–1.09	0.105
Other cutaneous type	0.46	0.24–0.90	0.023
Unknown subtype	0.50	0.31–0.83	0.008
Unknown primary	0.71	0.42–1.19	0.196
Age	1.01	1.00–1.02	<0.001
Prior treatment	1.14	1.02–1.27	0.015
LDH > ULN	1.04	0.93–1.16	0.474
BRAF mutation	1.38	1.04–1.84	0.028

## 4. Discussion

This is the first study to comprehensively assess whether primary tumor characteristics and initial presentation impacted the subsequent immunotherapy response in the metastatic setting. Though a more advanced stage of initial presentation, ulceration, and histologic subtype were associated with inferior outcomes in the univariate analyses, they were less or not significant in the multivariable analysis. Instead, cutaneous melanoma and each of its subtypes (superficial spreading, nodular, other, unknown) were significantly associated with improved clinical outcomes. Other features of the primary, including Breslow depth, TILs, mitoses, and LVI, did not impact the immunotherapy response after controlling for multiple variables.

There remains a need to identify the markers of response and survival to better select patients likely to benefit from ICI. The melanoma biology influences the immune response by regulating antigen expression and presentation, thereby playing a critical role in determining the likelihood of an immunotherapy response. The increased tumor cell expression of MHC-II and PD-L1, influenced by tumor infiltrating lymphocytes, has shown promise [19,20,21,22,23], although they remain imperfect [7,24,25]. A high tumor mutational burden (TMB) is a potential marker of response, although it has a limited predictive capacity [26,27]. Similarly, an inflamed tumor microenvironment, as expressed by CD8+ T cells and interferon-γ signatures, has also been correlated with a response to ICI therapies [28,29]. Therapeutic resistance has also been associated with transcriptional programs of mesenchymal differentiation, angiogenesis, and hypoxia, as well as JAK-STAT mutations [30,31]. While these biomarkers have promise, it is possible that the clinical and pathologic features of primary tumors could also have predictive value.

The ulceration and stage of presentation were associated with poorer PFS and OS in the univariate analyses, with similar but more modest effects seen with high mitotic rates, though these associations did not persist in the multivariable models. Thus, it is likely that factors like ulceration and a high mitotic rate are not only associated with higher rates of metastatic progression and death in the pre-ICI era [32] but also with other adverse metastatic features, such as visceral organ involvement, bulky disease, and high LDH, which correlate with ICI failure [33,34]. Indeed, ulceration was associated with higher metastatic stage at presentation with a marginally significant *p*-value, although not with higher LDH.

The lack of an independent association between most of the primary tumor features and outcomes following ICI therapy for metastatic disease suggests that these features may not have a major predictive role in this setting. However, they add valuable prognostic information following initial diagnosis. Since most of these adverse prognostic factors were identified prior to immunotherapy development, it is noteworthy that they presage not only inferior outcomes in an era lacking effective systemic therapies but also poor responses to active modern treatment options.

All of the cutaneous subtypes were more likely to respond compared with the acral melanoma, though “other” cutaneous subtypes (lentigo maligna, desmoplastic, spindle cell, etc.) had the longest PFS. Specifically, the ORR was greatest for the desmoplastic (N = 8, RR: 75%) and amelanotic subtypes (N = 3, RR: 67%), followed by lentigo maligna (N = 11, RR: 45%) and spindle cell (N = 8, RR: 38%). It is well known that the desmoplastic melanoma has a higher response in part due to its high mutation burden [35]. However, larger studies on less common atypical subtypes (such as amelanotic and lentigo maligna) Nare warranted to assess for other unexpected beneficial associations.

Several known prognostic factors were recapitulated. The metastatic stage (M1c, M1d) was associated with shorter PFS, while age and prior therapy correlated with shorter OS [36,37]. The *BRAF*^V600E^ mutation negatively impacted ICI response, possibly due to prior BRAF/MEK inhibitors in some patients. The optimal first-line regimen for BRAF-mutant melanomas remains contested, although it should consist of immunotherapy for most patients [38]. Ongoing studies aim to determine combination and sequence strategies (NCT02908672, NCT02967692, NCT02631447).

One prior study assessed primary tumor characteristics as potential biomarkers of immunotherapy response. LVI was significantly associated with an immunotherapy response and prolonged PFS and OS in advanced melanoma patients [39]. Our findings are somewhat in line with this, as LVI was associated with prolonged OS. Notably, our study has a significantly larger cohort and assessed multiple other clinical variables.

The limitations of this study included its retrospective and single-center nature, and relatively small sample size. Prospective, multi-center studies with a larger and more diverse sample size should be conducted to enhance generalizability.

These findings suggest that the clinical and pathological variables of primary tumors are associated with immunotherapy response, PFS, and OS in melanoma, though these do not appear to be independent of the adverse prognostic features of the metastatic presentation. Thus, primary features, other than cutaneous primary, should likely not be integrated in predictive models [40] for metastatic treatment, but may provide additional prognostic data surrounding initial presentation. Future research should aim to replicate and validate these results to help stratify patients by likelihood of response to ICI therapy.

## 5. Conclusions

Features of the primary tumor were associated with clinical outcomes when assessing as univariate analyses (primarily ulceration and initial stage of presentation). However, these associations were largely not significant after adjusting for known prognostic variables, with the exception of cutaneous histology vs. acral and mucosal primaries. This suggests that adverse prognostic features of initial presentation may correlate with later inferior outcomes with modern therapies, although these variables are captured with known prognostic features at the time of metastatic disease.

## Figures and Tables

**Table 1:** Primary tumor characteristics.

	PD-1 Monotherapy (N = 300)	Ipi/Nivo (N = 147)	Overall (N = 447)
Subtype			
Acral	22 (7.3%)	7 (4.8%)	29 (6.5%)
Cutaneous–superficial spreading	68 (22.7%)	39 (26.5%)	107 (23.9%)
Cutaneous–nodular	53 (17.7%)	23 (15.6%)	76 (17.0%)
Cutaneous–other	24 (8.0%)	5 (3.4%)	29 (6.5%)
Cutaneous–unknown	94 (31.3%)	32 (21.8%)	126 (28.2%)
Unknown primary	39 (13.0%)	41 (27.9%)	80 (17.9%)
Breslow thickness (mm)			
Mean (SD)	4.1 (4.7)	4.3 (6.4)	4.2 (5.3)
Median [Q1, Q3]	2.8 [1.5, 4.5]	2.1 [1.2, 5.8]	2.7 [1.4, 5.0]
n/a	86 (28.7%)	58 (39.5%)	144 (32.2%)
Ulceration			
0	96 (32.0%)	59 (40.1%)	155 (34.7%)
1	105 (35.0%)	32 (21.8%)	137 (30.6%)
n/a	99 (33.0%)	56 (38.1%)	155 (34.7%)
Mitoses (mm2)			
Mean (SD)	6.9 (7.4)	5.4 (4.6)	6.5 (6.7)
Median [Q1, Q3]	5.0 [2.0, 10.0]	4.0 [1.0, 8.0]	4.0 [2.0, 10.0]
n/a	143 (47.7%)	78 (53.1%)	221 (49.4%)
Lymphovascular invasion			
0	24 (8.0%)	10 (6.8%)	34 (7.6%)
1	118 (39.3%)	51 (34.7%)	169 (37.8%)
n/a	158 (52.7%)	86 (58.5%)	244 (54.6%)
Tumor-infiltrating lymphocytes			
0	46 (15.3%)	20 (13.6%)	66 (14.8%)
1	89 (29.7%)	39 (26.5%)	128 (28.6%)
n/a	165 (55.0%)	88 (59.9%)	253 (56.6%)
Number of positive sentinel nodes			
0	98 (32.7%)	56 (38.1%)	154 (34.5%)
1–2	76 (25.3%)	19 (12.9%)	95 (21.3%)
3+	21 (7.0%)	10 (6.8%)	31 (6.9%)
No SLN performed	105 (35.0%)	62 (42.2%)	167 (37.4%)
Stage at presentation			
1	46 (15.3%)	32 (21.8%)	78 (17.4%)
2	66 (22.0%)	31 (21.1%)	97 (21.7%)
3	125 (41.6%)	40 (27.2%)	165 (36.9%)
4	63 (21.0%)	44 (29.9%)	107 (23.9%)
M status at presentation			
M0	237 (79.0%)	102 (69.4%)	339 (75.8%)
M1a	18 (6.0%)	8 (5.4%)	26 (5.8%)
M1b	9 (3.0%)	2 (1.4%)	11 (2.5%)
M1c	26 (8.7%)	18 (12.2%)	44 (9.8%)
M1d	10 (3.3%)	17 (11.6%)	27 (6.0%)
Primary presentation			
Localized	124 (41.3%)	66 (44.9%)	190 (42.5%)
Locoregional	13 (4.3%)	9 (6.1%)	22 (4.9%)
Regional/Stage III	99 (33.0%)	26 (17.7%)	125 (27.9%)
Metastatic	64 (21.3%)	46 (31.3%)	110 (24.6%)
Missing	1 (0.3%)	1 (0.7%)	2 (0.4%)

This table demonstrates the primary tumor characteristics of the study patients.

**Table 2:** Confounding factors and outcomes of interest.

	PD-1 Monotherapy (N = 300)	Ipi/Nivo (N = 147)	Overall (N = 447)
Age			
Mean (SD)	62.6 (14.2)	57.2 (14.9)	60.9 (14.6)
Median [Q1, Q3]	64.0 [54.0, 73.0]	61.0 [47.0, 68.5]	63.0 [51.0, 72.0]
Gender			
Female	94 (31.3%)	43 (29.3%)	137 (30.6%)
Male	206 (68.7%)	104 (70.7%)	310 (69.4%)
Pre-immunotherapy treatment (metastatic)			
No	119 (39.7%)	87 (59.2%)	206 (46.1%)
Yes	181 (60.3%)	59 (40.1%)	240 (53.7%)
Missing	0 (0%)	1 (0.7%)	1 (0.2%)
LDH > ULN			
0	178 (59.3%)	64 (43.5%)	242 (54.1%)
1	107 (35.7%)	70 (47.6%)	177 (39.6%)
Missing	15 (5.0%)	13 (8.8%)	28 (6.3%)
BRAF V600E			
No	215 (71.7%)	91 (61.9%)	306 (68.5%)
Yes	84 (28.0%)	56 (38.1%)	140 (31.3%)
Missing	1 (0.3%)	0 (0%)	1 (0.2%)
Response to anti-PD-1			
PD/SD	172 (57.3%)	78 (53.1%)	250 (55.9%)
CR/PR	128 (42.7%)	69 (46.9%)	197 (44.1%)
Missing with PD/SD	3 (1.0%)	10 (6.8%)	13 (2.9%)
Progressed			
0	93 (31.0%)	52 (35.4%)	145 (32.4%)
1	207 (69.0%)	95 (64.6%)	302 (67.6%)
PFS (months)			
Mean (SD)	19.3 (26.5)	20.2 (25.0)	19.6 (26.0)
Median [Q1, Q3]	7.4 [2.6, 23.0]	7.2 [2.4, 30.5]	7.4 [2.5, 26.2]
Died			
0	125 (41.7%)	75 (51.0%)	200 (44.7%)
1	175 (58.3%)	72 (49.0%)	247 (55.3%)
OS (months)			
Mean (SD)	31.1 (30.9)	28.7 (25.1)	30.3 (29.1)
Median [Q1, Q3]	19.5 [8.3, 48.7]	22.2 [7.7, 44.8]	19.8 [8.0, 48.2]

This table demonstrates the confounding factors in the study patients and their outcomes of interest.

**Table 3:** Univariate analysis: response to anti-PD-1, PFS, and OS.

Response to Anti-PD-1		
Variable	*p*-value	FDR adjusted *p*-value
Subtype	0.001	0.003
Breslow thickness (mm)	0.327	0.545
Ulceration	0.009	0.023
Mitoses (mm2)	0.527	0.589
Lymphovascular invasion	0.476	0.589
Tumor-infiltrating lymphocytes	1	1
Number of positive sentinel nodes	0	0.003
Stage at presentation	0.008	0.023
M status at presentation	0.53	0.589
Primary presentation	0.03	0.06
BRAF mutation	<0.001	<0.001
PFS *		
Variable	*p*-value	FDR adjusted *p*-value
Subtype	0.004	0.012
Breslow thickness (mm)	0.889	0.889
Ulceration	0.002	0.01
Mitoses (mm2)	0.07	0.116
Lymphovascular invasion	0.093	0.133
Tumor-infiltrating lymphocytes	0.316	0.351
Number of positive sentinel nodes	0.001	0.008
Stage at presentation	0.046	0.114
M status at presentation	0.31	0.351
Primary presentation	0.059	0.116
BRAF mutation	<0.001	<0.001
OS *		
Variable	*p*-value	FDR adjusted *p*-value
Subtype	0.21	0.262
Breslow thickness (mm)	0.641	0.641
Ulceration	0.039	0.118
Mitoses (mm2)	0.059	0.118
Lymphovascular invasion	0.566	0.629
Tumor-infiltrating lymphocytes	0.098	0.141
Number of positive sentinel nodes	0.004	0.04
Stage at presentation	0.095	0.141
M status at presentation	0.024	0.118
Primary presentation	0.059	0.118
BRAF mutation	0.19	0.257

* For continuous variables, the values are categorized into quantiles for the log-rank test. (*p*-values and FDR-adjusted *p*-values for log-rank tests of clinical variables by PFS).

## Data Availability

The raw data supporting the conclusions of this article will be made available by the authors on reasonable request.
